# The balancing act: Identifying multivariate sports performance using Pareto frontiers

**DOI:** 10.3389/fspor.2022.918946

**Published:** 2022-08-04

**Authors:** Tim Newans, Phillip Bellinger, Clare Minahan

**Affiliations:** ^1^Griffith Sports Science, Griffith University, Gold Coast, QLD, Australia; ^2^Queensland Academy of Sport, Nathan, QLD, Australia

**Keywords:** cricket, visualization, talent identification, optimal selection, front

## Abstract

Athletes often require a mix of physical, physiological, psychological, and skill-based attributes that can be conflicting when competing at the highest level within their sport. When considering multiple variables in tandem, Pareto frontiers is a technique that can identify the observations that possess an optimal balance of the desired attributes, especially when these attributes are negatively correlated. This study presents Pareto frontiers as a tool to identify athletes who possess an optimal ranking when considering multiple metrics simultaneously. This study explores the trade-off relationship between batting average and strike rate as well as bowling strike rate, economy, and average in Twenty 20 cricket. Eight hundred ninety-one matches of Twenty 20 cricket from the men's (MBBL) and women's (WBBL) Australian Big Bash Leagues were compiled to determine the best batting and bowling performances, both within a single innings and across each player's Big Bash career. Pareto frontiers identified 12 and seven optimal batting innings performances in the MBBL and WBBL respectively, with nine and six optimal batting careers respectively. Pareto frontiers also identified three optimal bowling innings in both the MBBL and WBBL and five and six optimal bowling careers in MBBL and WBBL, respectively. Each frontier identified players that were not the highest ranked athlete in any metric when analyzed univariately. Pareto frontiers can be used when assessing talent across multiple metrics, especially when these metrics may be conflicting or uncorrelated. Using Pareto frontiers can identify athletes that may not have the highest ranking on a given metric but have an optimal balance across multiple metrics that are associated with success in a given sport.

## Introduction

Many sports require athletes to possess well-developed physiological or mechanical characteristics (e.g., endurance, maximal sprint speed) and/or skill-related attributes (i.e., shot speed and shot accuracy) that are often opposing and/or are not associated. These disparate attributes appear to be more obvious between athletes in team sports when compared to specific disciplines in individual sports that require highly specific attributes for success [e.g., track sprint cycling (Lievens et al., 2021) or marathon running (Jones et al., 2021)]. Furthermore, it is not unusual for team sports to include both specialists, such as athletes who possess superior maximal sprint speed and athletes who possess superior endurance capacity, and individual athletes who have a wide range of physiological/mechanical and skill-related attributes. For instance, in stick or racquet sports such as golf and tennis, each athlete is required to balance the accuracy and speed of their strokes (Wells et al., 2009; Maquirriain et al., 2016); while in most team sports, an athlete should develop a balance of speed and endurance (Stølen et al., 2005). Moreover, in sports like cricket, an athlete is required to balance their batting/bowling average with their batting/bowling strike rate or economy (Barr and Kantor, 2004; Patel et al., 2017). In each of these examples, a case could be made for which is the preferred attribute; however, the preferred attribute may differ given the other athletes within a particular team or given a particular situation within a single match. A cohesive team may require a squad of players that differ in their balance of attributes and, therefore, it is apparent that performance analysis requires a multi-faceted approach when selecting prospective players.

While talent identification processes have been extensively reported (Falk et al., 2004; Pyne et al., 2005; Pion et al., 2015; Till et al., 2016; Dodd and Newans, 2018; Johnston et al., 2018), standards and benchmarks are typically reported univariately; that is, each attribute is assessed in isolation. For example, players could be standardized within each attribute (i.e., z-scores) to determine where the athlete sits with respect to the rest of the athletic population (Turner et al., 2019). However, this method has a flaw, in that some attributes are negatively correlated, as well as physiological/mechanical characteristics such as maximal sprint speed and endurance capacity (Sánchez-García et al., 2018). Therefore, if an athlete excels in one attribute, it is likely that this would come at the expense of a conflicting attribute. By assessing talent identification univariately, athletes are identified when they have specialist skills (e.g., strongest, fastest, fittest, leanest etc.) (Minahan et al., 2021). Although a given sport often requires a balance of these attributes, it is reasonable to suggest that talent identification processes should assess talent multivariately, that is, multiple variables in tandem, rather than univariately.

The process of optimizing the balance of multiple attributes is termed “multi-objective optimization”. This technique is becoming increasingly of interest with recent developments in machine learning algorithms; however, their origins are quite simple mathematically in that they aim to create the perfect balance of the attributes of interest. If a data point was defined as: $\vec{x_{1}}\;\epsilon \;X$, it is, therefore, better than another data point defined by: $\vec{x_{2}}\;\epsilon \;X$ if $f_{i}\left(\vec{x_{1}}\right)\;\leq \;f_{i}\left(\vec{x_{2}}\right)\;$for all metrics *i ϵ* {1, 2, …, *k*} and $f_{j}\left(\vec{x_{1}}\right)<f_{j}\left(\vec{x_{2}}\right)\;$for at least one metric *j ϵ* {1, 2, …, *k*}. Once these conditions have been met, the remaining points are deemed Pareto-optimal and form what is called the Pareto frontier. There are two key strengths to using Pareto frontiers. Firstly, when balancing multiple attributes, Pareto-optimal observations can be identified with just few lines of computer code. Secondly, when visualizing a limited number of attributes (i.e., three or less), the Pareto frontier is intuitive and can be clearly identified, assisting in the translation and interpretation of the results to coaches and other support staff. This has been used in other fields such as designing aircrafts with maximum aerodynamic efficiency, maximum range, and minimum weight (Mastroddi and Gemma, 2013). However, there is very limited use of Pareto frontiers within sport (Perez-Toledano et al., 2019). By using Pareto frontiers, the optimal balance of all these attributes can be identified rather than guessing through siloed univariate analyses.

To illustrate the concept of Pareto frontiers, the present study used batting and bowling data in Twenty 20 (T20) cricket. Like other forms of cricket (i.e., Test and one-day matches), T20 cricket requires players to score as many runs as possible within the allotted 20 overs without being dismissed (i.e., being bowled, caught out, run out etc.). In addition, players of T20 cricket also need to score runs in as few deliveries as possible. Therefore, it is difficult to determine whether 80 runs “off” [i.e., from] 60 balls or 40 runs off 20 balls is of more benefit to a team as their differing risk profiles contribute differently to the formation of the team (Bukiet and Ovens, 2006). For example, early on in an innings the risk-return of attempting to hit six runs off a ball is significantly different than in the final over of an innings. Similarly, a bowler needs to balance taking wickets while also conceding as few runs as possible. For instance, when bowling four overs, it is again difficult to determine whether taking three wickets for 50 runs is of more worth than taking no wickets but only conceding eight runs as the three wickets may not have been worth conceding 50 runs. Therefore, when assessing the quality of players, it is necessary to utilize tools that can analyse these datasets without favoring one metric over another. The concept of Pareto frontiers is one such tool and, therefore, the present study aimed to introduce Pareto frontiers to the sports science community and illustrate how they can identify players with the optimal balance of attributes that can be obfuscated when performing univariate analysis across each metric.

## Materials and methods

The present study comprised all 489 matches of the first 11 editions of the Men's Big Bash League (MBBL) and all 402 matches of the first seven editions of the Women's Big Bash League (WBBL), Australia's domestic T20 cricket competition. The MBBL dataset contained 423 batters and 313 bowlers, while the WBBL dataset contained 214 batters and 159 bowlers. All scorecards were freely available online. Collectively, there were 13,764 individual batting innings with observations ranging from 1 to 113 innings per batter, while there were 10,796 individual bowling innings with observations ranging from 1 to 106 innings per bowler.

To summarize the data, two summary statistics were generated for batting and three summary statistics were generated for bowling. The summary statistics were as follows:

- Batting Average: runs scored divided by frequency of dismissal
- Batting Strike Rate: runs scored divided by balls faced multiplied by 100
- Bowling Average: runs conceded divided by wickets taken
- Bowling Strike Rate: balls bowled divided by wickets taken
- Bowling Economy: runs conceded divided by overs (i.e., 6 balls) bowled

To understand optimal batting performance, the number of runs scored as well as the rate at which these runs were scored are both of importance in Twenty20 cricket (Barr and Kantor, 2004). As it was expected that there would be a trade-off relationship between these variables, there would be multiple batters that possess an optimal balance of these attributes. Therefore, it was deemed appropriate that a Pareto frontier could be established to identify these batters. Similarly, to identify optimal bowling performance, the number of wickets, runs conceded, and rate of which the wickets and runs are recorded are all of importance (Patel et al., 2017). As the number of wickets taken can come at the expense of runs conceded, it was also expected that no bowler would be optimal in every attribute and therefore, a Pareto frontier would also be required to identify the bowlers that possess the optimal balance of these bowling attributes.

Consequently, four Pareto frontiers for each competition were established within the dataset:

- *Pareto-optimal Batting Innings*
- This analysis outlined the highest runs scored within an innings at the highest strike rate.
- *Pareto-optimal Batting Career*
- This analysis outlined the highest batting average across a career at the highest strike rate. To provide a more accurate career report, batters required to have played a minimum of 15 innings which left 158 eligible male batters and 116 eligible female batters.
- *Pareto-optimal Bowling Innings*
- This analysis outlined the most wickets taken in an innings at the lowest economy.
- *Pareto-optimal Bowling Career*
- This analysis outlined the lowest bowling average across a career at the lowest economy and lowest strike rate. To provide a more accurate career report, bowlers required to have bowled a minimum of 200 balls which left 137 eligible male bowlers and 98 eligible female bowlers.

All data was analyzed using R (v 4.1.0) statistical software (R Core Team., 2019) with the dataset and script attached as Supplementary Files. Firstly, the *dplyr* (Wickham et al., 2021) and *tidyr* (Wickham, 2021) packages were used for data manipulation to format the data into the correct structure to identify the Pareto frontiers (see Line 10 of the attached script for an example). The *rPref* package (Roocks, 2016) was used to determine the Pareto frontiers using the *psel* function with the “top_level” argument set to 999 to ensure every athlete was assigned to a frontier (see Line 15 of the attached script for an example). Once the frontiers were established, the *ggplot2* (Wickham, 2016) and *scatterplot3D* (Ligges and Mächler, 2003) packages were used to visualize the data and subsequent Pareto frontiers (see Lines 17 and 125 of the attached script for respective examples).

## Results

### Men's Pareto-optimal batting

**Figure 1:**
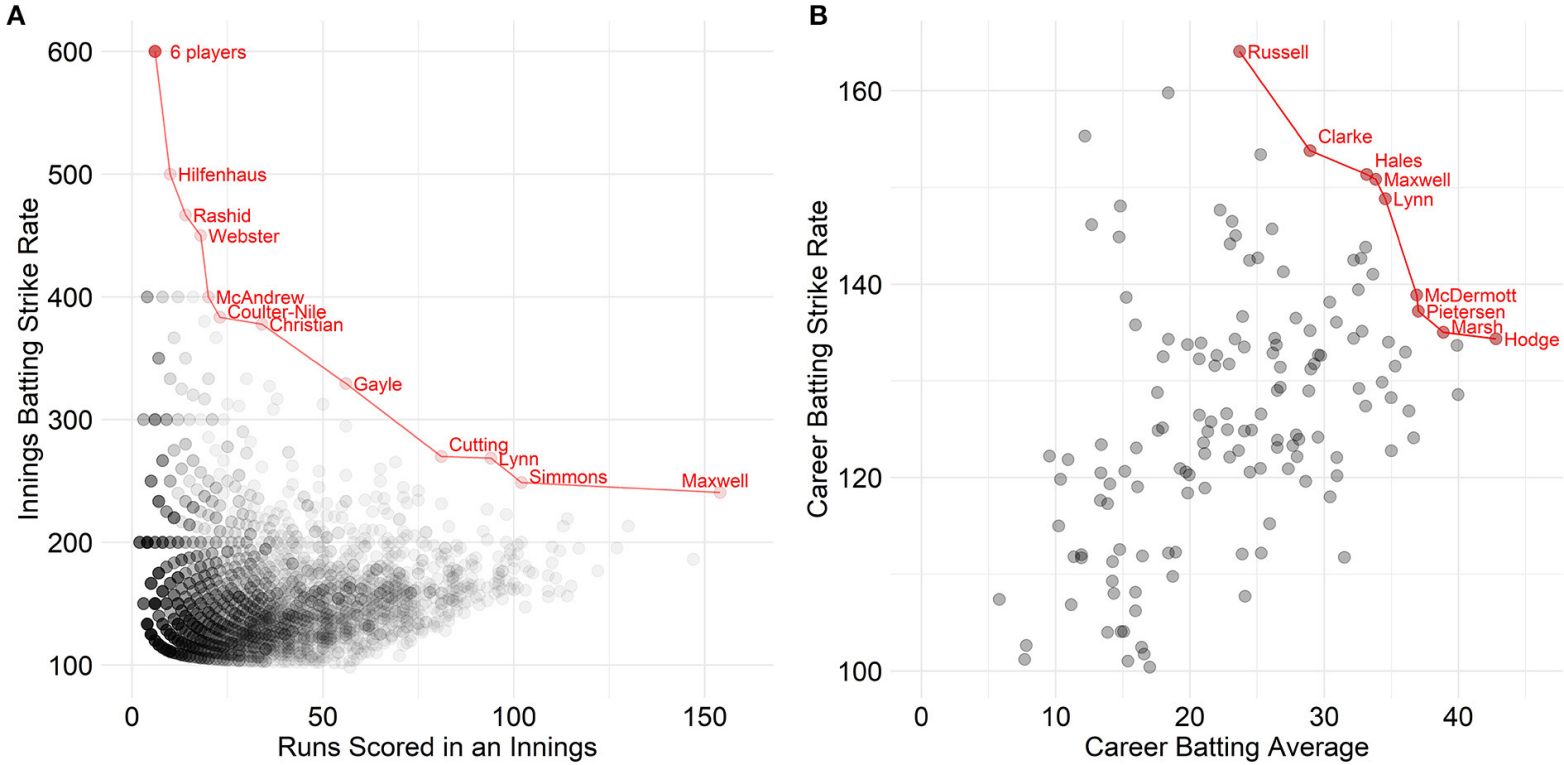
Men's Pareto-optimal batting within an innings **(A)** and across a career **(B)** with the Pareto frontier highlighted in red. N.B. For illustrative purposes, points were filtered out in A if both their runs scored was below 50 and their strike rate was below 100 and points were filtered out in B if both their average was below 20 and their strike rate was below 100.

As seen by the red line in Figure 1A, 11 Pareto-optimal innings were identified as Pareto-optimal innings. That is, no other batter has scored more runs at a faster strike rate than these 12 innings. These innings ranged from 6 runs off 1 ball (i.e., strike rate = 600) to 154 off 64 balls (i.e., strike rate = 240.63). Additionally, the solution of 6 runs off 1 ball has been attained six times. In Figure 1B, nine Pareto-optimal batting careers were identified (in red) with Andre Russell achieving the highest strike rate (164.07) and Brad Hodge achieving the highest average (42.78), while Joe Clarke, Alex Hales, Glenn Maxwell, Chris Lynn, Ben McDermott, Kevin Pietersen and Mitchell Marsh were all deemed Pareto-optimal due to varying combinations of both metrics.

### Men's Pareto-optimal bowling

**Figure 2:**
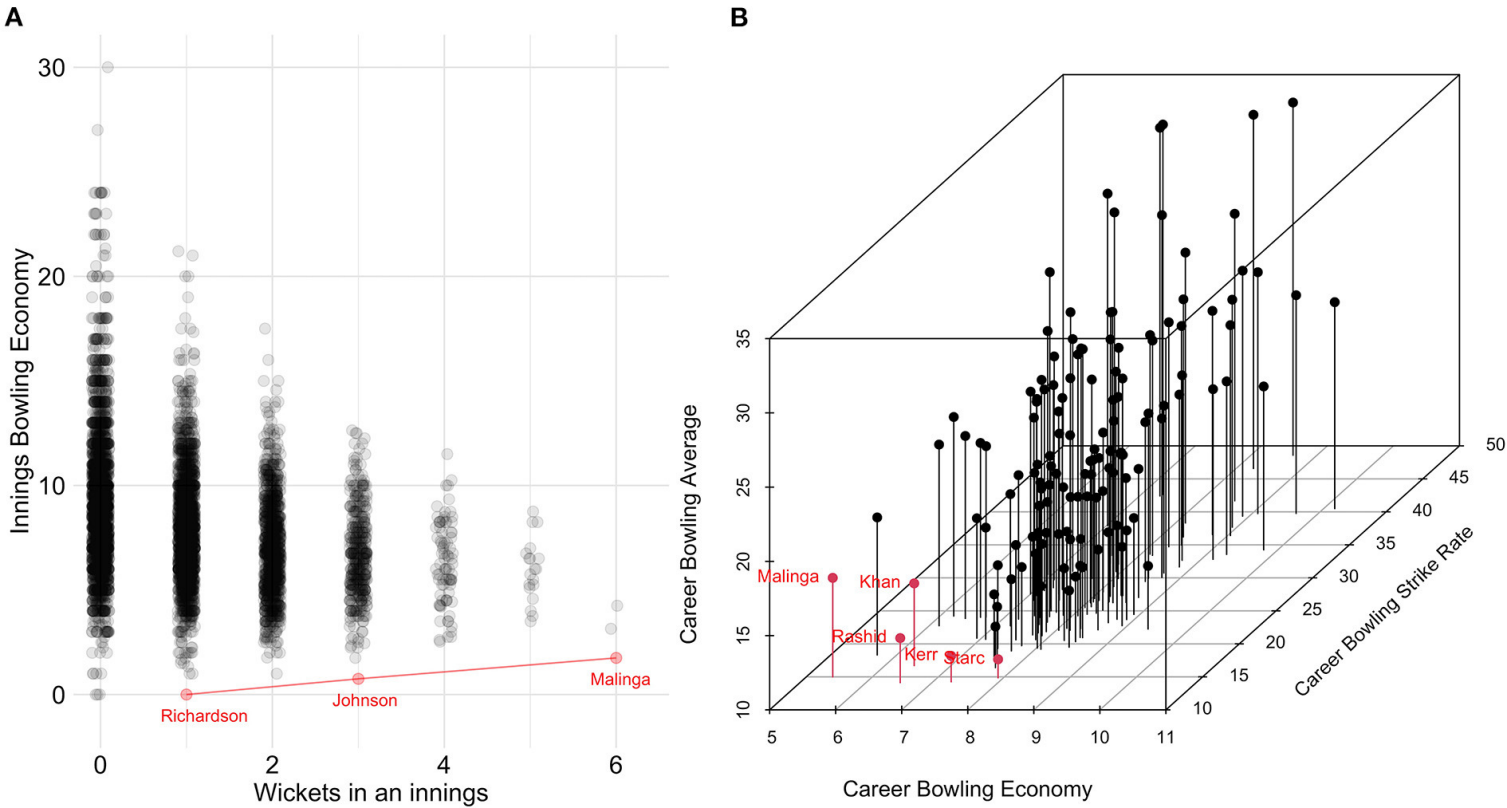
Men's Pareto-optimal bowling within an innings **(A)** and across a career **(B)** with the points on the Pareto frontier highlighted in red.

Three Pareto-optimal bowling innings in Figure 2A were identified: 1/0 (i.e., 1 wicket for 0 runs conceded) by Jhye Richardson, 3/3 by Mitchell Johnson, and 6/7 achieved by Lasith Malinga. While there were three occurrences of 0/0, none of these are deemed Pareto-optimal as 1/0 by Richardson supersedes this combination. Five Pareto-optimal bowling careers were identified in Figure 2B as having the best balance of bowling average, strike rate, and economy, with Adil Rashid achieving the lowest average (14.13), Lasith Malinga achieving the lowest economy (5.40), Mitchell Starc achieving the lowest strike rate (11.25), while Rashid Khan and Hayden Kerr were deemed Pareto-optimal due to a combination of the three metrics.

### Women's Pareto-optimal batting

**Figure 3:**
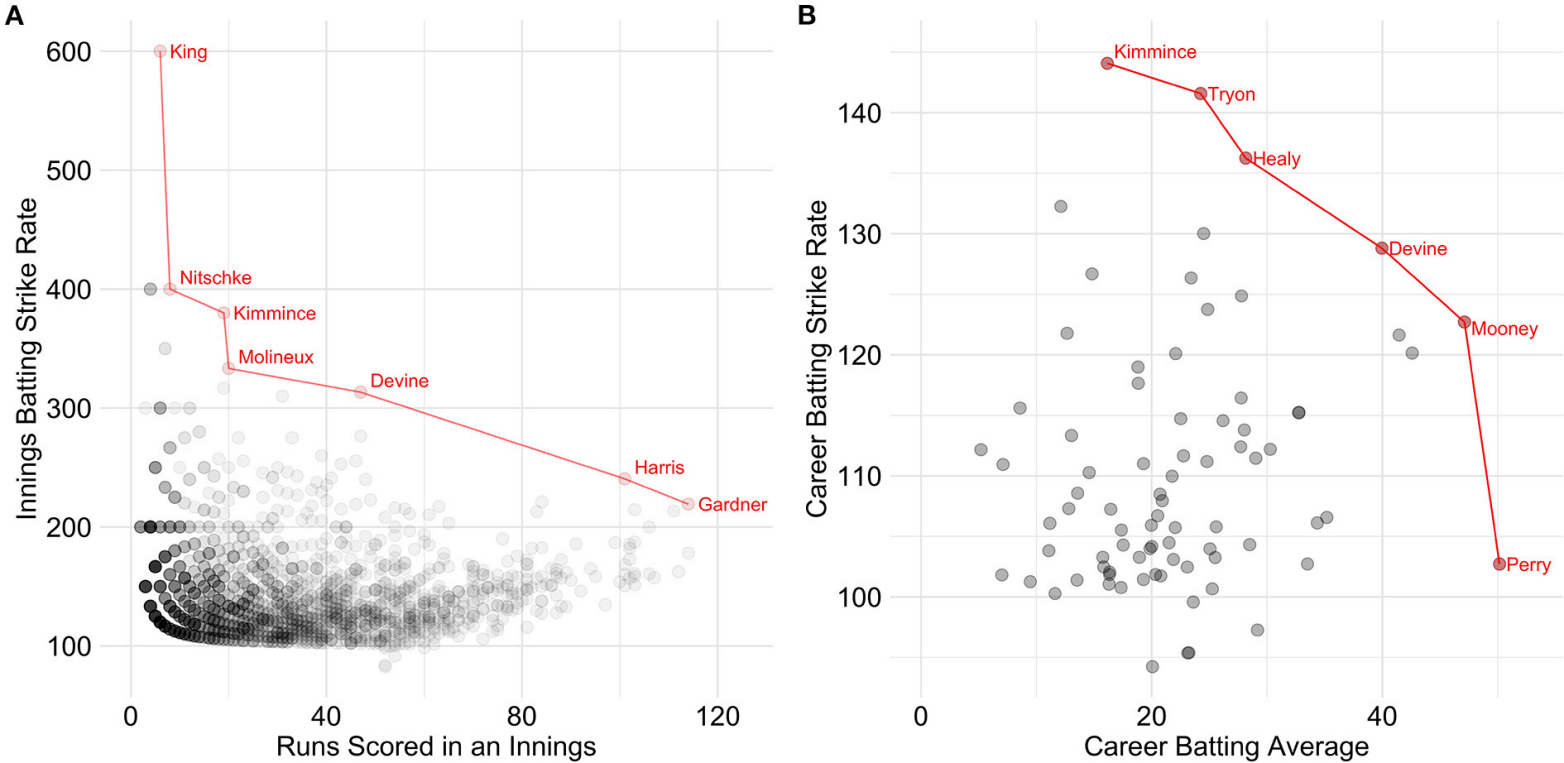
Women's Pareto-optimal batting within an innings **(A)** and across a career **(B)** with the Pareto frontier highlighted in red. N.B. For illustrative purposes, points were filtered out in A if both their runs scored was below 50 and their strike rate was below 100 and points were filtered out in B if both their average was below 20 and their strike rate was below 100.

Seven Pareto-optimal innings were identified in Figure 3A with extremities ranging from 6 runs off 1 ball (i.e., strike rate = 600) to 114 off 52 balls (i.e., strike rate = 219.23). In Figure 3B, six Pareto-optimal batting careers were also identified with Laura Kimmince achieving the highest strike rate (144.08), Ellyse Perry achieving the highest average (50.15), with other Pareto-optimal solutions due to varying combinations of both metrics.

### Women's Pareto-optimal bowling

**Figure 4:**
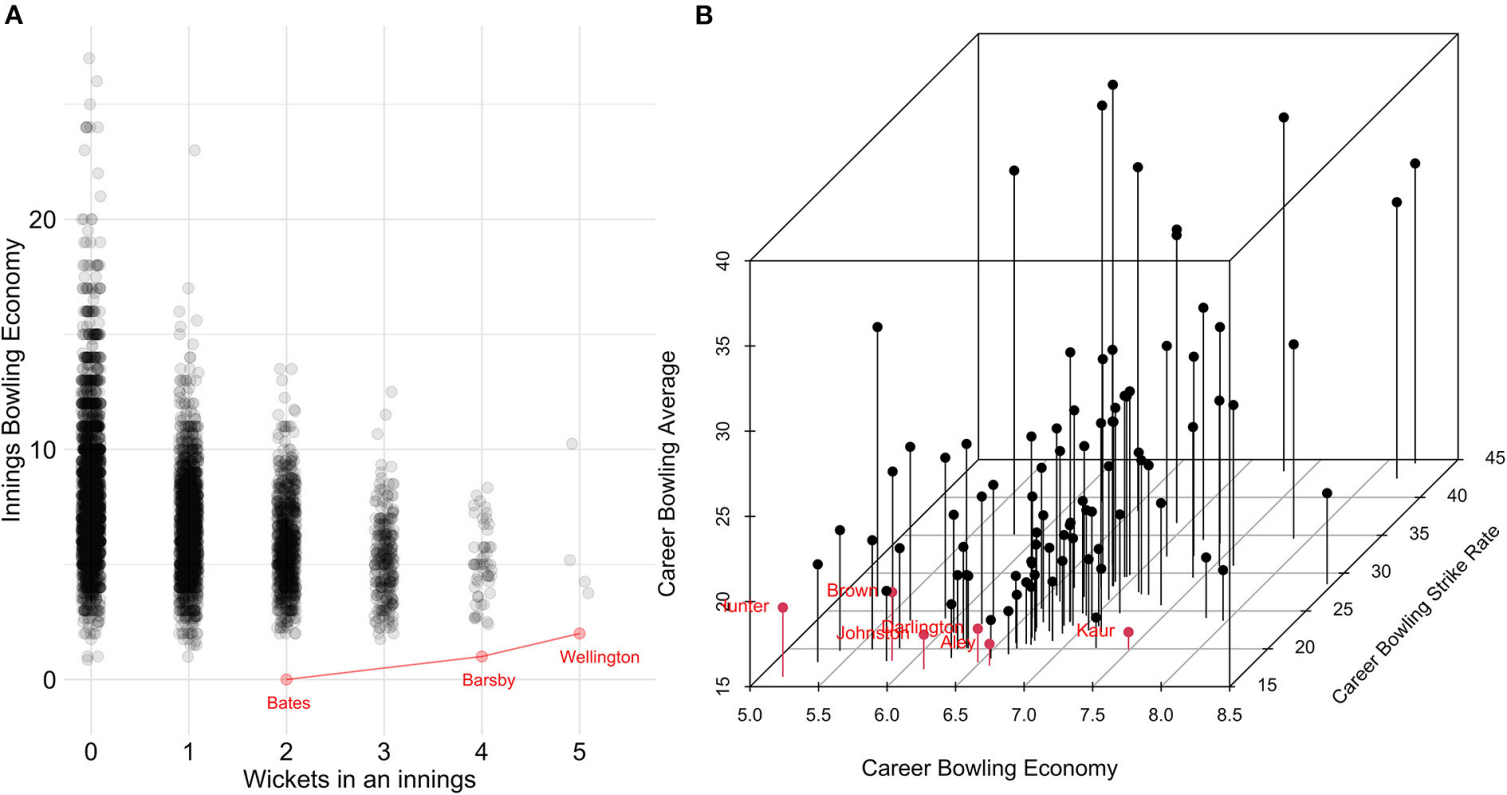
Women's Pareto-optimal bowling within an innings **(A)** and across a career **(B)** with the points on the Pareto frontier highlighted in red.

Three Pareto-optimal bowling innings in Figure 4A were identified: 2/0 by Samantha Bates, 4/2 by Jemma Barsby, and 5/8 achieved by Amanda-Jade Wellington. Six Pareto-optimal bowling careers were identified in Figure 4B as having the best balance of bowling average, strike rate, and economy, with Julie Hunter achieving both the lowest average (16.38) and lowest economy (5.16), Harmanpreet Kaur achieving the lowest strike rate (16.00), while Sarah Aley, Darcie Brown, Ruth Johnston, and Hannah Darlington are deemed Pareto-optimal due to a combination of the three metrics.

## Discussion

The present study aimed to introduce Pareto frontiers to sports scientists and its application in identifying and visualizing the most extraordinary players when considering multiple variables. While it is intuitively recognized that multiple attributes are required for success in sport, by identifying the Pareto frontier between these attributes is a simple, yet effective method to identify all the players that possess an optimal balance of these attributes relative to other individuals in the cohort. By analyzing talent multivariately, rather than simply analyzing multiple variables univariately, players can be deemed optimal despite not being objectively highest in a single variable. The present study highlights that when there are conflicting attributes that are of equal interest, the attributes should be viewed in tandem using Pareto frontiers, or else there is a risk that the expectations of an individual to attain the highest level in both attributes univariately may be unfeasible. This was evident in all eight Pareto frontiers, as at least one athlete was identified in each example that was not identified as highest ranked athlete in any metric when analyzed univariately, and yet was deemed Pareto-optimal due to their balance in the metrics of interest. For instance, when observing the career batting average Pareto frontier in the WBBL (Figure 3B), there is an expansive continuum of batters that are all deemed Pareto-optimal as they each have slightly different average/strike rate profiles.

The main advantage of Pareto frontiers highlighted in the present study is identifying athletes who are optimal across multiple metrics even when they are not the highest ranked in any metric. This was most evident in the MBBL where, Chris Lynn when viewed univariately, has the 14th-highest career batting average (34.54), which is 8.24 runs per innings lower than the highest (Figure 1B). Similarly, he has the eighth-highest strike rate, striking at 148.84 which is 15.22 runs per 100 balls lower than the highest. However, when considering both metrics simultaneously and visualizing these metrics, it is clear that he is one of the best batters across the 11 seasons of the MBBL.

Pareto frontiers can also be used to provide benchmarks when conflicting attributes are both desirable and by analyzing these attributes in tandem, more realistic expectations can be set for each athlete depending on whether they sit in the cartesian plane. The resulting benchmarks can be more individualized than what can be expected when viewing metrics univariately. Consequently, by viewing these metrics multivariately, different levels of quality within differing squad roles can be better expressed. Pareto frontiers can be used not only for extreme values, but can also be used to visualize the second, third, and n-th frontiers to further interrogate where an individual lies within the cartesian space. For example, in Figure 3B, it was evident that there is a substantial gap between the first and second WBBL career batting Pareto frontiers.

In this study we chose to observe batting and bowling as purely independent roles within cricket; however, there are also avenues for Pareto frontiers to be established for all-rounders within cricket (i.e., players that are picked for both their batting and bowling ability). However, it should be noted that if an all-rounder Pareto frontier were to be established with both batting average and strike rate as well as bowling average, economy, and strike rate, the resulting five-dimensional outputs, while valid and executable, become increasingly difficult to interpret and visualize. To do such an analysis, a factor-reduction technique such as principal components analysis should be considered and the Pareto frontier could be built from the extracted components (e.g., batting and bowling).

This technique could also be used to identify “maximal” efforts when multiple observations of an individual are recorded which is common in sports science practice. For example, (Duthie et al., 2021) sought to define maneuverability by identifying the maximum tortuosity within each 0.5 m·s^−1^ increment of speed and plotting the line of best fit through these points (Duthie et al., 2021). In this instance, a Pareto frontier could have been fitted to the data to eliminate the need for an arbitrary selection of 0.5 m·s^−1^ speed bins. Similarly, Morin et al. (2021) sought to develop an *in-situ* acceleration-speed profile by identifying the maximum acceleration within each 0.2 m·s^−1^ of speed and plotting the line of best fit through these points (Morin et al., 2021). By using a Pareto frontier, the need for an arbitrating 3 m·s^−1^ threshold and 0.2 m·s^−1^ speed bins could have been avoided. Finally, Rudsits et al. (2018) sought to identify the torque-cadence and power-cadence profile of cyclists by identifying maximal torque or power values in each 5 rpm bin (Rudsits et al., 2018). If a Pareto frontier were used, the model would not require any additional filtering to remove “non-maximal” efforts as the frontier will have already deemed those points non-optimal.

The present study also illustrated how Pareto frontiers can be used to visualize talent in more than 2 dimensions. For example, while Darcie Brown has the seventh-lowest bowling average, 11th-lowest economy, and the 18th-lowest strike rate (see Figure 4B), she can be deemed a Pareto-optimal bowler as there are no other bowlers who supersede her across all three metrics. Similarly, in the MBBL (Figure 2B), while Rashid Khan has the sixth-lowest average, sixth-lowest economy and the 18th-lowest strike rate, he can be deemed a Pareto-optimal bowler as there are no other bowlers who supersede him across all three metrics. While there will be some correlations between the three bowling metrics (i.e., average, economy, and strike rate) as the metrics are related (e.g., wickets taken is the denominator of average and numerator of strike rate), visualizing the third dimension is still necessary as the reader would still need to multiply the x and y values to understand where they would sit in the third dimension. This could be further expanded into higher dimensions; however, these dimensions become increasingly difficult to visualize.

It should also be considered that there is some level of uncertainty surrounding each observation in the career Pareto frontiers due to the differing number of observations. For example, Joe Clarke is deemed Pareto-optimal as he is currently striking at 153.82 at an average of 28.94 after 16 innings; however, it is right to assume that it is more uncertain that he lies on the frontier than Chris Lynn who has 100 observations. Therefore, future research could consider providing confidence or credible intervals around the probability that an individual lies on the Pareto frontier. Consequently, it is then feasible that a probability that an individual sits on the first, second, or third frontier could be calculated.

While the present study used Twenty20 cricket to illustrate the power and usefulness of Pareto frontiers, the concept can be widely applied within sports science datasets, especially when the variables of interest are uncorrelated or negatively correlated. Pareto frontiers can still be established between two positively correlated metrics; however, it is likely that there will be less “hidden” athletes on this frontier as naturally the athletes who are high in one metric will be high in the other metric. Future research should apply Pareto frontiers across different avenues within sports performance analysis, such as repeated-sprint ability and dynamic strength index which have multi-faceted determinants. In addition, there are many other possibilities within sport science whereby Pareto frontiers can reveal athletes who possess the optimal balance of the metrics of interest.

## Conclusion

With the proliferation of various physiological, mechanical, and skill-related attributes associated with performance, Pareto frontiers should be used within sports science to visualize multiple performance metrics. By analyzing opposing data in tandem, more feasible expectations and benchmarks can be established to reveal talent that may have been missed when analyzing multiple metrics univariately.

## Data availability statement

The original contributions presented in the study are included in the article/Supplementary material, further inquiries can be directed to the corresponding author.

## Ethics statement

Ethical review and approval was not required for the study on human participants in accordance with the local legislation and institutional requirements. Written informed consent for participation was not required for this study in accordance with the national legislation and the institutional requirements.

## Author contributions

TN: project concept, data collection and analysis, and preparation of manuscript. PB and CM: project concept, refining, and synthesizing manuscript. All authors contributed to the article and approved the submitted version.

## Conflict of interest

The authors declare that the research was conducted in the absence of any commercial or financial relationships that could be construed as a potential conflict of interest. The reviewer HT declared a past co-authorship with the authors CM to the handling editor.

## Publisher's note

All claims expressed in this article are solely those of the authors and do not necessarily represent those of their affiliated organizations, or those of the publisher, the editors and the reviewers. Any product that may be evaluated in this article, or claim that may be made by its manufacturer, is not guaranteed or endorsed by the publisher.

## References

[B1] Barr G. D. I. Kantor B. S. A. (2004). criterion for comparing and selecting batsmen in limited overs cricket. J. Oper. Res. Soc. 55, 1266–1274. 10.1057/palgrave.jors.2601800

[B2] Bukiet B. Ovens M. (2006). A mathematical modelling approach to one-day cricket batting orders. J. Sports. Sci. Med. 5, 495–502. Available online at: www.jssm.org/24357943 PMC3861747

[B3] Dodd K. D. Newans T. J. (2018). Talent identification for soccer: Physiological aspects. J. Sci. Med. Sport. 21, 1073–1078. 10.1016/j.jsams.2018.01.009 29789264

[B4] Duthie G. M. Robertson S. Thornton H. R. A. (2021). GNSS-based method to define athlete manoeuvrability in field-based team sports. PloS ONE. 16, e0260363. 10.1371/journal.pone.0260363 34797902 PMC8604331

[B5] Falk B. Lidor R. Lander Y. Lang B. (2004). Talent identification and early development of elite water-polo players: a 2-year follow-up study. J. Sports Sci. 22, 347–355. 10.1080/02640410310001641566 15161108

[B6] Johnston K. Wattie N. Schorer J. Baker J. (2018). Talent identification in sport: a systematic review. Sports Med. 48, 97–109. 10.1007/s40279-017-0803-2 29082463

[B7] Jones A. M. Kirby B. S. Clark I. E. Rice H. M. Fulkerson E. Wylie L. J.. (2021). Physiological demands of running at 2-hour marathon race pace. J. Appl. Physiol. 130, 369–379. 10.1152/japplphysiol.00647.2020 33151776

[B8] Lievens E. Bellinger P. Van Vossel K. Vancompernolle J. Bex T. Minahan C.. (2021). Muscle typology of world-class cyclists across various disciplines and events. Med. Sci. Sports Exerc. 53, 816–824. 10.1249/MSS.0000000000002518 33105386

[B9] Ligges U. Mächler M. (2003). Scatterplot3d - an R package for visualizing multivariate data. J. Stat. Softw. 8, 1–20. 10.18637/jss.v008.i11

[B10] Maquirriain J. Baglione R. Cardey M. (2016). Male professional tennis players maintain constant serve speed and accuracy over long matches on grass courts. Eur. J. Sport Sci. 16, 845–849. 10.1080/17461391.2016.1156163 26960753

[B11] Mastroddi F. Gemma S. (2013). Analysis of Pareto frontiers for multidisciplinary design optimization of aircraft. Aerosp. Sci. Technol. 28, 40–55. 10.1016/j.ast.2012.10.003

[B12] Minahan C. Newans T. Quinn K. Parsonage J. Buxton S. Bellinger P. (2021). Strong, Fast, fit, lean, and safe: a positional comparison of physical and physiological qualities within the 2020 Australian women's rugby league team. J. Strength Cond. Res. 35, S11–S19. 10.1519/JSC.0000000000004106 34319942

[B13] Morin J-B. Le Mat Y. Osgnach C. Barnabo A. Pilati A.. (2021). Individual acceleration-speed profile in-situ: a proof of concept in professional football players. J. Biomech. 123, 110524. 10.1016/j.jbiomech.2021.110524 34023754

[B14] Patel A. K. Bracewell P. J. Gazley A. J.. (2017). Identifying fast bowlers likely to play test cricket based on age-group performances. Int. J. Sports Sci. Coach. 12, 328–338. 10.1177/1747954117710514

[B15] Perez-Toledano M. A. Rodriguez F. J. Garcia-Rubio J. Ibanez S. J. (2019). Players' selection for basketball teams, through Performance Index Rating, using multiobjective evolutionary algorithms. PloS ONE. 14, e0221258. 10.1371/journal.pone.0221258 31483835 PMC6726145

[B16] Pion J. Lenoir M. Vandorpe B. Segers V. (2015). Talent in female gymnastics: a survival analysis based upon performance characteristics. Int. J. Sports Med. 94, 935–940. 10.1055/s-0035-1548887 26212248

[B17] Pyne D. B. Gardner A. S. Sheehan K. Hopkins W. G. (2005). Fitness testing and career progression in AFL football. J. Sci. Med. Sport. 8, 321–332. 10.1016/S1440-2440(05)80043-X 16248473

[B18] R Core Team. (2019). R: A Language and Environment for Statistical Computing. Vienna, Austria: R Foundation for Statistical Computing. Available online at: https://www.R-project.org/

[B19] Roocks P. (2016). Computing Pareto Frontiers and Database Preferences with the rPref Package. R. J. 8, 393–404. 10.32614/RJ-2016-054

[B20] Rudsits B. L. Hopkins W. G. Hautier C. A. Rouffet D. (2018). Force-velocity test on a stationary cycle ergometer: methodological recommendations. J. Appl. Physiol. 124, 831–839. 10.1152/japplphysiol.00719.2017 29357495

[B21] Sánchez-García M. Sánchez-Sánchez J. Rodríguez-Fernández A. Solano D. Castillo D. (2018). Relationships between sprint ability and endurance capacity in soccer referees. Sports. 6, 28. 10.3390/sports6020028 29910332 PMC6027545

[B22] Stølen T. Chamari K. Castagna C. Wisløff U. (2005). Physiology of soccer: an update. Sports Med. 35, 501–536. 10.2165/00007256-200535060-00004 15974635

[B23] Till K. Cobley S. Morley D. (2016). O'hara J, Chapman C, Cooke C. The influence of age, playing position, anthropometry and fitness on career attainment outcomes in rugby league. J. Sports Sci. 34, 1240–1245. 10.1080/02640414.2015.1105380 26512761

[B24] Turner A. N. Jones B. Stewart P. Bishop C. Parmar N. Chavda S.. (2019). Total score of athleticism: holistic athlete profiling to enhance decision-making. Strength Cond. J. 41, 91–101. 10.1519/SSC.0000000000000506

[B25] Wells G. D. Elmi M. Thomas S. (2009). Physiological correlates of golf performance. J. Strength Cond. Res. 23, 741–750. 10.1519/JSC.0b013e3181a07970 19387406

[B26] Wickham H. (2016). ggplot2: Elegant Graphics for Data Analysis. Springer-Verlag New York. Available online at: https://ggplot2.tidyverse.org 10.1007/978-3-319-24277-4_9

[B27] Wickham H. (2021). tidyr: Tidy Messy Data. Available online at: https://CRAN.R-project.org/package=tidyr

[B28] Wickham H. François R. Henry L. Müller K. (2021). dplyr: A Grammar of Data Manipulation. Available online at: https://CRAN.R-project.org/package=dplyr

