# Supplementary material for: The balancing act: Identifying multivariate sports performance using Pareto frontiers
Source: Front Sports Act Living. 2022 Aug 4;4:918946. doi: 10.3389/fspor.2022.918946 (PMC9386182; doi:10.3389/fspor.2022.918946)
Supplement: Supplementary file 5 [file Data_Sheet_5.DOCX]

## Load in Packages

{ library(dplyr)

library(tidyr)

library(ggplot2)

library(rPref)

library(scatterplot3d)

library(patchwork)

}

# MEN'S BATTING

mbblbat <- read.csv('mbblbat.csv') # Men's batting scorecards

mbblbat <- mbblbat %>%

mutate(SR = R/B*100) %>%

separate(Batter,c("FirstName","LastName"), sep = " ", extra = "merge", remove = F)

## Men's Pareto Batting Innings

BatInnPareto_men <- psel(mbblbat %>% filter(R > 0),high(R)*high(SR),top_level = 999)

(MenBatInnParetoPlot <- ggplot(mapping = aes(x = R, y = SR)) +

geom_point(data = BatInnPareto_men %>% filter(R > 50 | SR > 100), alpha=0.05, size = 3) +

geom_point(data = BatInnPareto_men %>% filter(.level == 1), alpha = 0.1, color = "red",size = 3)+

geom_text(data = BatInnPareto_men %>% filter(.level == 1 & R != 6 & !(LastName %in% c("Coulter-Nile","Cutting","Simmons","Maxwell"))), aes(label = LastName), color = "Red", hjust = "left",nudge_x = 2)+

geom_text(data = BatInnPareto_men %>% filter(.level == 1 & LastName %in% c("Coulter-Nile","Simmons")), aes(label = LastName), color = "Red", hjust = "left",nudge_x = 2,nudge_y = 5)+

geom_text(data = BatInnPareto_men %>% filter(.level == 1 & LastName == "Cutting"), aes(label = LastName), color = "Red", hjust = "left",nudge_x = 2,nudge_y = 10)+

geom_text(data = BatInnPareto_men %>% filter(.level == 1 & LastName == "Maxwell"), aes(label = LastName), color = "Red", hjust = "left",nudge_x = -10,nudge_y = 10)+

geom_line(data = BatInnPareto_men %>% filter(.level == 1), alpha = 0.5, colour = "red")+

annotate(geom = "text",x = 20,y = 600, label = "6 players",color = "red")+

scale_y_continuous(breaks = seq(100,600,100))+

coord_cartesian(xlim = c(0,160))+

theme_minimal() +

labs(x = "Runs Scored in an Innings",

y = "Innings Batting Strike Rate") +

theme(axis.title = element_text(size = 16),

panel.grid.minor.y = element_blank(),

axis.text = element_text(size = 16, color = "black")))

## Men's Pareto Batting Career

{dismissals_men <- mbblbat %>%

group_by(Batter) %>%

filter(isOut == T) %>%

summarise(Dismissals = n())

notouts_men <- mbblbat %>%

group_by(Batter) %>%

filter(isOut == F) %>%

summarise(NotOuts = n())

sumBat_men <- mbblbat %>%

group_by(Batter,LastName) %>%

summarise(TotalRuns = sum(R), TotalBalls = sum(B), Innings = n()) %>%

ungroup()

sumBat_men <- left_join(sumBat_men,dismissals_men)

sumBat_men <- left_join(sumBat_men,notouts_men)

sumBat_men$Dismissals[is.na(sumBat_men$Dismissals)] <- 0

sumBat_men$NotOuts[is.na(sumBat_men$NotOuts)] <- 0

sumBat_men <- sumBat_men %>%

mutate(Average = TotalRuns/Dismissals,

StrikeRate = TotalRuns/TotalBalls*100)

filtBat_men <- sumBat_men %>%

filter(Innings >= 15)

}

BatCarPareto_men <- psel(filtBat_men,high(Average)*high(StrikeRate),top_level = 999) %>%

filter(Average > 20 | StrikeRate > 100)

(MenBatCarParetoPlot <- ggplot(BatCarPareto_men, aes(x = Average, y = StrikeRate)) +

geom_point(alpha=0.3, size = 3) +

geom_point(data = BatCarPareto_men %>% filter(.level == 1), alpha = 0.3, color = "red",size = 3)+

geom_line(data = BatCarPareto_men %>% filter(.level == 1), color = "red")+

geom_text(data = BatCarPareto_men %>% filter(.level == 1 & LastName == "Clarke"), aes(label = LastName), color = "red", hjust = "left",nudge_x = 0.6,nudge_y = 1.5)+

geom_text(data = BatCarPareto_men %>% filter(.level == 1 & LastName == "Hales"), aes(label = LastName), color = "red", hjust = "left",nudge_x = 0.6,nudge_y = 1.5)+

geom_text(data = BatCarPareto_men %>% filter(.level == 1 & LastName == "Marsh"), aes(label = LastName), color = "red", hjust = "left",nudge_x = 0.6,nudge_y = 0.6)+

geom_text(data = BatCarPareto_men %>% filter(.level == 1 & !LastName %in% c("Clarke","Hales","Marsh")), aes(label = LastName), color = "red", hjust = "left",nudge_x = 0.6)+

theme_minimal() +

coord_cartesian(xlim = c(0,45.5))+

labs(x = "Career Batting Average",

y = "Career Batting Strike Rate") +

theme(axis.title = element_text(size = 16),

legend.position = "none",

axis.text = element_text(size = 16, color = "black")))

MenBatPlot <- MenBatInnParetoPlot + MenBatCarParetoPlot

MenBatPlot + plot_annotation(tag_levels = 'A')

ggsave("Figure 1.tiff", height = 6.5, width = 13, dpi = 600)

# MEN'S BOWLING

mbblbowl <- read.csv('mbblbowl.csv') # Men's bowling scorecards

mbblbowl <- mbblbowl %>%

mutate(Econ = R/Balls*6) %>%

separate(Bowling,c("FirstName","LastName"), extra = "merge")

## Men's Pareto Bowling Innings

BowlInnPareto_men <- psel(mbblbowl,high(W)*low(Econ),top_level = 999)

(MenBowlInnParetoPlot <- ggplot(BowlInnPareto_men, aes(x = W, y = Econ)) +

geom_jitter(data = BowlInnPareto_men %>% filter(.level != 1),aes(x = W, y = Econ), alpha=0.1, size = 3, width = 0.1) +

geom_point(data = BowlInnPareto_men %>% filter(.level == 1), aes(x = W, y = Econ), alpha = 0.3, shape = 21, size = 3, fill = "red", color = "red") +

geom_text(data = BowlInnPareto_men %>% filter(.level == 1), aes(x = W, y = Econ, label = LastName), color = "Red", hjust = "left",nudge_x = -0.3,nudge_y = -1)+

geom_line(data = BowlInnPareto_men %>% filter(.level == 1), aes(x = W, y = Econ), alpha = 0.5, colour = "red")+

theme_minimal() +

labs(x = "Wickets in an innings",

y = "Innings Bowling Economy") +

coord_cartesian(xlim = c(0,6.3))+

theme(axis.title = element_text(size = 16),

legend.position = "none",

panel.grid.minor.y = element_blank(),

axis.text = element_text(size = 16, color = "black")))

## Men's Pareto Bowling Career

{sumBowl_men <- mbblbowl %>%

group_by(id) %>%

summarise(Innings = n(),Balls = sum(Balls), Wickets = sum(W), Runs = sum(R)) %>%

mutate(Average = Runs/Wickets,

Economy = Runs/Balls*6,

StrikeRate = Balls/Wickets)

mbblbowlernames <- mbblbowl %>% distinct(id,FirstName,LastName)

filtBowl_men <- sumBowl_men %>%

left_join(mbblbowlernames) %>%

filter(Balls >= 200) %>%

ungroup()

}

BowlCarPareto_men <- psel(filtBowl_men,low(Average)*low(StrikeRate)*low(Economy),top_level = 999) %>%

filter(Average < 50)

BowlCarPareto_men$color <- case_when(BowlCarPareto_men$.level == 1 ~ 2,

BowlCarPareto_men$.level > 1 ~ 1)

BowlCarPareto_men$Label[BowlCarPareto_men$.level == 1] <- BowlCarPareto_men$LastName[BowlCarPareto_men$.level == 1]

MenBowlCarParetoPlot <-scatterplot3d(BowlCarPareto_men[c("Economy","Average","StrikeRate")], type = "h",pch = 16, color=BowlCarPareto_men$color,

xlab="Career Bowling Economy",

ylab="Career Bowling Strike Rate",

zlab="Career Bowling Average")

zz.coords <- MenBowlCarParetoPlot$xyz.convert(BowlCarPareto_men$Economy, BowlCarPareto_men$Average, BowlCarPareto_men$StrikeRate)

text(zz.coords$x,

zz.coords$y,

labels = BowlCarPareto_men$Label,

cex = .8,

pos = 2,

col = "red")

# WOMEN'S BATTING

wbblbat <- read.csv('wbblbat.csv') # Women's batting scorecards

wbblbat <- wbblbat %>%

mutate(SR = R/B*100) %>%

separate(Batter,c("FirstName","LastName"), extra = "merge", remove = F)

## Women's Pareto Batting Innings

BatInnPareto_women <- psel(wbblbat %>% filter(R >0),high(R)*high(SR),top_level = 999)

(WomenBatInnParetoPlot <- ggplot(BatInnPareto_women %>% filter(R > 50 | SR > 100), aes(x = R, y = SR)) +

geom_point(alpha=0.05, size = 3) +

geom_point(data = BatInnPareto_women %>% filter(.level == 1), alpha = 0.1, color = "red",size = 3)+

geom_text(data = BatInnPareto_women %>% filter(.level == 1 & !(LastName %in% c("Nitschke","Molineux","Devine","Harris"))), aes(label = LastName), color = "Red", hjust = "left",nudge_x = 2)+

geom_text(data = BatInnPareto_women %>% filter(.level == 1 & LastName == "Nitschke"), aes(label = LastName), color = "Red", hjust = "left",nudge_x = 2,nudge_y = 10)+

geom_text(data = BatInnPareto_women %>% filter(.level == 1 & LastName == "Molineux"), aes(label = LastName), color = "Red", hjust = "left",nudge_x = 2,nudge_y = 10)+

geom_text(data = BatInnPareto_women %>% filter(.level == 1 & LastName == "Devine"), aes(label = LastName), color = "Red", hjust = "left",nudge_x = 2,nudge_y = 10)+

geom_text(data = BatInnPareto_women %>% filter(.level == 1 & LastName == "Harris"), aes(label = LastName), color = "Red", hjust = "left",nudge_x = 2,nudge_y = 10)+

geom_line(data = BatInnPareto_women %>% filter(.level == 1), alpha = 0.5, colour = "red")+

scale_y_continuous(breaks = seq(100,600,100))+

coord_cartesian(xlim = c(0,125))+

theme_minimal() +

labs(x = "Runs Scored in an Innings",

y = "Innings Batting Strike Rate") +

theme(axis.title = element_text(size = 16),

panel.grid.minor.y = element_blank(),

axis.text = element_text(size = 16, color = "black")))

## Women's Pareto Batting Career

{dismissals_women <- wbblbat %>%

group_by(id) %>%

filter(isOut == T) %>%

summarise(Dismissals = n())

notouts_women <- wbblbat %>%

group_by(id) %>%

filter(isOut == F) %>%

summarise(NotOuts = n())

sumBat_women <- wbblbat %>%

group_by(id) %>%

summarise(TotalRuns = sum(R), TotalBalls = sum(B), Innings = n()) %>%

ungroup()

sumBat_women <- left_join(sumBat_women,dismissals_women)

sumBat_women <- left_join(sumBat_women,notouts_women)

sumBat_women$Dismissals[is.na(sumBat_women$Dismissals)] <- 0

sumBat_women$NotOuts[is.na(sumBat_women$NotOuts)] <- 0

sumBat_women <- sumBat_women %>%

mutate(Average = TotalRuns/Dismissals,

StrikeRate = TotalRuns/TotalBalls*100)

wbblbatternames <- wbblbat %>% distinct(id,FirstName,LastName)

filtBat_women <- sumBat_women %>%

left_join(wbblbatternames) %>%

filter(Innings >= 15)

}

BatCarPareto_women <- psel(filtBat_women,high(Average)*high(StrikeRate),top_level = 999)%>%

filter(Average > 20 | StrikeRate > 100)

(WomenBatCarParetoPlot <- ggplot(BatCarPareto_women, aes(x = Average, y = StrikeRate)) +

geom_point(alpha=0.3, size = 3) +

geom_point(data = BatCarPareto_women %>% filter(.level == 1), alpha = 0.3, color = "red",size = 3)+

geom_line(data = BatCarPareto_women %>% filter(.level == 1), color = "red")+

geom_text(data = BatCarPareto_women %>% filter(.level == 1 & !LastName %in% c("Kimmince","Lanning","Mooney")), aes(label = LastName), color = "Red", hjust = "left",nudge_x = 0.6)+

geom_text(data = BatCarPareto_women %>% filter(.level == 1 & LastName == "Kimmince"), aes(label = LastName), color = "Red", hjust = "left",nudge_x = 0.6,nudge_y = 1)+

geom_text(data = BatCarPareto_women %>% filter(.level == 1 & LastName == "Lanning"), aes(label = LastName), color = "Red", hjust = "left",nudge_x = 0.6,nudge_y = 1.5)+

geom_text(data = BatCarPareto_women %>% filter(.level == 1 & LastName == "Mooney"), aes(label = LastName), color = "Red", hjust = "left",nudge_x = 0.6,nudge_y = -0.5)+

coord_cartesian(xlim = c(0,53))+

theme_minimal() +

labs(x = "Career Batting Average",

y = "Career Batting Strike Rate") +

theme(axis.title = element_text(size = 16),

legend.position = "none",

axis.text = element_text(size = 16, color = "black")))

# WOMEN'S BOWLING

wbblbowl <- read.csv('wbblbowl.csv') # Women's bowling scorecards

wbblbowl <- wbblbowl %>%

mutate(Econ = R/Balls*6) %>%

separate(Bowling,c("FirstName","LastName"), sep = " ",extra = "merge")

## Women's Pareto Bowling Innings

BowlInnPareto_women <- psel(wbblbowl,high(W)*low(Econ),top_level = 999)

(WomenBowlInnParetoPlot <- ggplot(BowlInnPareto_women, aes(x = W, y = Econ)) +

geom_jitter(data = BowlInnPareto_women %>% filter(.level != 1),aes(x = W, y = Econ), alpha=0.1, size = 3, width = 0.1) +

geom_point(data = BowlInnPareto_women %>% filter(.level == 1), aes(x = W, y = Econ), alpha = 0.3, shape = 21, size = 3, fill = "red", color = "red") +

geom_text(data = BowlInnPareto_women %>% filter(.level == 1), aes(x = W, y = Econ, label = LastName), color = "Red", hjust = "left",nudge_x = -0.2,nudge_y = -1)+

geom_line(data = BowlInnPareto_women %>% filter(.level == 1), aes(x = W, y = Econ), alpha = 0.5, colour = "red")+

theme_minimal() +

coord_cartesian(xlim = c(0,5.5))+

scale_x_continuous(breaks = c(0:5))+

labs(x = "Wickets in an innings",

y = "Innings Bowling Economy") +

theme(axis.title = element_text(size = 16),

legend.position = "none",

panel.grid.minor.x = element_blank(),

axis.text = element_text(size = 16, color = "black")))

## Women's Pareto Bowling Career

{sumBowl_women <- wbblbowl %>%

group_by(FirstName, LastName) %>%

summarise(Innings = n(),Balls = sum(Balls), Wickets = sum(W), Runs = sum(R)) %>%

mutate(Average = Runs/Wickets,

Economy = Runs/Balls*6,

StrikeRate = Balls/Wickets)

filtBowl_women <- sumBowl_women %>%

filter(Balls >= 200) %>%

ungroup()

}

BowlCarPareto_women <- psel(filtBowl_women,low(Average)*low(StrikeRate)*low(Economy),top_level = 999) %>%

filter(Average < 50)

BowlCarPareto_women$color <- case_when(BowlCarPareto_women$.level == 1 ~ 2,

BowlCarPareto_women$.level > 1 ~ 1)

BowlCarPareto_women$Label[BowlCarPareto_women$.level == 1] <- BowlCarPareto_women$LastName[BowlCarPareto_women$.level == 1]

WomenBowlCarParetoPlot <-scatterplot3d(BowlCarPareto_women[c("Economy","Average","StrikeRate")], type = "h",pch = 16, color=BowlCarPareto_women$color,

xlab="Career Bowling Economy",

ylab="Career Bowling Strike Rate",

zlab="Career Bowling Average")

zz.coords <- WomenBowlCarParetoPlot$xyz.convert(BowlCarPareto_women$Economy, BowlCarPareto_women$Average, BowlCarPareto_women$StrikeRate)

text(zz.coords$x,

zz.coords$y,

labels = BowlCarPareto_women$Label,

cex = .8,

pos = 2,

col = "red")

MenBatPlot <- MenBatInnParetoPlot + MenBatCarParetoPlot

MenBatPlot + plot_annotation(tag_levels = 'A')

ggsave("Figure 1.tiff", height = 6, width = 6*1.75, dpi = 600)

MenBowlInnParetoPlot

ggsave("Figure 2A.png", height = 7, width = 6, dpi = 600)

png("Figure 2B.png", height = 7, width = 7, units = "in", res = 600)

MenBowlCarParetoPlot <-scatterplot3d(BowlCarPareto_men[c("Economy","Average","StrikeRate")], type = "h",pch = 16, color=BowlCarPareto_men$color,

xlab="Career Bowling Economy",

ylab="Career Bowling Strike Rate",

zlab="Career Bowling Average")

zz.coords <- MenBowlCarParetoPlot$xyz.convert(BowlCarPareto_men$Economy, BowlCarPareto_men$Average, BowlCarPareto_men$StrikeRate)

text(zz.coords$x,

zz.coords$y,

labels = BowlCarPareto_men$Label,

cex = .8,

pos = 2,

col = "red")

dev.off()

BatPlot <- (MenBatInnParetoPlot + MenBatCarParetoPlot) / (WomenBatInnParetoPlot + WomenBatCarParetoPlot)

BatPlot + plot_annotation(tag_levels = 'A')

ggsave("Figure 1jsams.tiff", height = 13, width = 13, dpi = 600)

WomenBowlInnParetoPlot

ggsave("Figure 4A.png", height = 7, width = 6, dpi = 600)

png("Figure 4B.png", height = 7, width = 7, units = "in", res = 1000)

WomenBowlCarParetoPlot <-scatterplot3d(BowlCarPareto_women[c("Economy","Average","StrikeRate")], type = "h",pch = 16, color=BowlCarPareto_women$color,

xlab="Career Bowling Economy",

ylab="Career Bowling Strike Rate",

zlab="Career Bowling Average")

zz.coords <- WomenBowlCarParetoPlot$xyz.convert(BowlCarPareto_women$Economy, BowlCarPareto_women$Average, BowlCarPareto_women$StrikeRate)

text(zz.coords$x,

zz.coords$y,

labels = BowlCarPareto_women$Label,

cex = .8,

pos = 2,

col = "red")

dev.off()
